# Evaluation of Autism Spectrum Disorder Risk in Infants With Intraventricular Hemorrhage

**DOI:** 10.7759/cureus.45541

**Published:** 2023-09-19

**Authors:** Irfan Shehzad, Muppala Raju, Ineshia Jackson, Madhava Beeram, Vinayak Govande, Arpitha Chiruvolu, Niraj Vora

**Affiliations:** 1 Neonatology, Baylor Scott & White Health, Temple, USA; 2 Neonatology, Baylor University Medical Center, Dallas, USA

**Keywords:** nicu, cranial ultrasound, mchat-r/f, intraventricular hemorrhage, autism spectrum disorder

## Abstract

Background

This study evaluates the long-term risk of autism spectrum disorder (ASD) in infants with intraventricular hemorrhage (IVH) using the Modified Checklist for Autism in Toddlers-Revised with Follow-Up (M-CHAT-R/F) screening tool.

Methods

This retrospective cohort study compared IVH (exposed) infants across all gestational age groups with no-IVH (non-exposed) infants admitted to level IV neonatal intensive care unit (NICU). The M-CHAT-R/F screening tool was used to assess the ASD risk at 16-30 months of age. Discharge cranial ultrasound (CUS) findings also determined the ASD risk. Descriptive statistics comprised median and interquartile range for skewed continuous data and frequencies and percentages for categorical variables. Comparisons for non-ordinal categorical measures in bivariate analysis were carried out using the χ*2* test or Fisher exact test.

Results

Of the 334 infants, 167 had IVH, and 167 had no IVH. High ASD risk (43% vs. 20%, p = 0.044) and cerebral palsy (19% vs. 5%, p = 0.004) were significantly associated with severe IVH. Infants with CUS findings of periventricular leukomalacia had 3.24 odds of developing high ASD risk (odds ratios/OR: 3.24, 95% confidence interval/CI: 0.73-14.34), and those with hydrocephalus needing ventriculoperitoneal (VP) shunt had 4.75 odds of developing high ASD risk (OR: 4.75, 95% CI: 0.73-30.69).

Conclusion

Severe IVH, but not mild IVH, increased the risk of ASD and cerebral palsy. This study demonstrates the need for timely screening for ASD in high-risk infants. Prompt detection leads to earlier treatment and better outcomes.

## Introduction

Autism spectrum disorder (ASD) is a biologically based neurodevelopmental disorder characterized by persistent deficits in social communication; social interaction; and restricted, repetitive patterns of behavior, interests, and activities [1,2]. ASD affects more than five million Americans, with an estimated prevalence of approximately 1.7% in children [1]. The prevalence of ASD is almost four times higher in infants born preterm compared to those born at term [3]. ASD has been proposed to be associated with congenital factors, such as genomic mutations [4,5]; maternal risk factors, such as mental illness, epilepsy, obesity, hypertension, diabetes, polycystic ovary syndrome, infection, asthma, assisted fertility, hyperemesis, and younger maternal age; and neonatal risk factors such as birth asphyxia, prematurity, and low gestational age [6].

Various neonatal morbidities may contribute to the increased risk of ASD. Among these complications are forms of perinatal brain injury such as intraventricular hemorrhage (IVH), parenchymal lesions that reflect focal ischemic or hemorrhagic injury to white matter, and ventricular enlargement that are detectable with neonatal cranial ultrasound (CUS) [7]. Although magnetic resonance imaging (MRI) is more sensitive than CUS for detecting white matter damage, CUS is safer, more efficient, and significantly less expensive than MRI [8]. The specific brain abnormalities on routine CUS in low gestational age infants are associated with cerebral palsy and other neurodevelopmental impairments [9]. The frequency of IVH among preterm infants ranges from 14.7% to 50%, with considerable variation across gestational age groups [10]. Severe IVH, mainly Papile’s grades 3 and 4, is associated with significant neurodevelopmental impairment in about 43% of affected infants [11].

It has been reported that intensive early intervention programs can improve cognitive and language abilities and adaptive behavior in children with ASD [12]. The American Academy of Pediatrics (AAP) recommends screening all children for symptoms of ASD through a combination of developmental surveillance at all primary care visits and standardized autism-specific screening tests at 18 and 24 months of age during their visits. Children with ASD can be identified as toddlers, and early intervention can improve outcomes [13]. Parent-completed questionnaires are the most common screening tests used in primary care. The Modified Checklist for Autism in Toddlers (M-CHAT) is the most studied and widely used tool for screening toddlers for ASD [14,15].

This article was previously posted to the ResearchSquare preprint server on August 29, 2022.

## Materials and methods

Study design and population

This retrospective cohort study was conducted on infants admitted to level IV neonatal intensive care unit (NICU) from January 2014 through June 2020, with follow-up in the primary care clinic through December 2021. The local institutional review board approved it. This study considered IVH as an exposure, and the outcome was to determine the relative risk of developing ASD in those exposed to IVH versus those not exposed (no-IVH). Those born with multiple congenital anomalies died or were transferred out before NICU discharge, and those with missing M-CHAT-R/F screening results were excluded.

Mild IVH is referred to as Grades I and II, and severe IVH is called Grades III and IV, as described by Papile et al. [16] on CUS. He described unilateral/bilateral germinal matrix hemorrhage for Grade I, IVH without ventricular dilatation for Grade II, IVH with ventricular dilatation for Grade III, and IVH extending into adjacent brain parenchyma for Grade IV.

Data was obtained from the hospital’s electronic medical record (EMR) system. Maternal data, including age, ethnicity, insurance, mode of delivery, pre-eclampsia, diabetes, antenatal steroids, antenatal magnesium, and psychiatric disorders, were obtained. Infant data including sex, gestational age, birth weight, intubation at resuscitation, Apgar scores at 1 and 5 minutes, postnatal systemic steroids, duration of mechanical ventilation, length of NICU stay, ventriculoperitoneal (VP) shunt placement, CUS findings of parenchymal lesions, IVH, periventricular leukomalacia (PVL), hydrocephalus, patent ductus arteriosus (PDA), severe retinopathy of prematurity (ROP), hearing screen failure at NICU discharge, and home discharge summary on oxygen monitor were reported. M-CHAT-R/F results, cerebral palsy, and speech or language delays were obtained from chart review and ICD9/10 codes.

Based on the established clinical practice guidelines at our institution, infants ≤ 32 weeks of gestation undergo at least two CUS: first, within 10 days of birth, mostly around seven days of life, and again at or about 36 weeks postmenstrual age (PMA) or around the time of NICU discharge. Infants born over 32 weeks of gestation undergo CUS as an aspect of diagnostic workup and clinical care (patients with seizure, hypoxic-ischemic injury, suspected brain hemorrhage, or infection). All CUS were performed by experienced technologists using high-frequency transducers and included multiple standard quasi-coronal and parasagittal views using the anterior fontanel as the sonographic window. These ultrasounds were interpreted by at least one pediatric radiologist trained in reading neonatal CUS to characterize the types and locations of abnormal findings of IVH, parenchymal lesions, PVL, and hydrocephalus with detailed specifications for severity.

Infants were screened by primary care providers for ASD risk using the Modified Checklist for Autism in Toddlers-Revised with Follow-Up (M-CHAT-R/F) questionnaire between 16 and 30 months of chronological age. Infants who have moderate (M-CHAT-R/F score ≥ 3-7) to high risk (M-CHAT-R/F score ≥ 8) at first screening were administered the same questionnaire at their follow-up/second screening visit. Infants who scored ≤2 were considered to pass or have a low risk for ASD, and those who scored ≥8 or continued to have ≥3-7 on follow-up/second screening were considered to fail or have a high risk of ASD or another developmental disorder.

Statistical analysis

Descriptive statistics included a median and interquartile range for skewed continuous variables and frequencies and percentages for categorical variables. For bivariate analysis, comparisons for non-ordinal categorical measures were conducted using χ^2^ or Fisher exact test, while comparisons for nonsymmetrical continuous variables were performed using the Wilcoxon rank sum test. Logistic regression was conducted while controlling for gestational age and birth weight to examine factors associated with ASD risk. These results were reported as odds ratios (ORs) with 95% confidence intervals (CIs). Data analysis was done by Stata 15.1 (StataCorp LLC, College Station, Texas). A p-value of <0.05 was considered statistically significant.

## Results

For this study, 334 infants were eligible, out of which 167 infants have IVH (exposed) and 167 have no IVH (non-exposed). Of these infants, M-CHAT-R/F screening test results were found in 80 infants in the IVH group and 85 infants in the no-IVH group at 16-30 months of chronological age. The final sample comparing high ASD risk to low ASD risk included 165 infants, with 35 infants in the high ASD risk group who failed M-CHAT-R/F and 130 infants in the low ASD risk group who passed M-CHAT-R/F.

Median gestational age and weight at birth were statistically significant between the high ASD risk and low ASD risk groups (25.6 vs. 29.4 weeks, p = 0.01 and 0.90 vs. 1.18 kg, p = 0.03). Statistically significant differences were also observed in receiving postnatal systemic steroids (60% vs. 25%, p = 0.0009), IVH (60% vs. 45%, p = 0.006), cerebral palsy (26% vs. 3%, p < 0.0001), speech delay (57% vs. 31%, p = 0.004), and severe ROP (49% vs. 21%, p = 0.012). Infants in the high ASD risk group were sicker, required longer lengths of stay in the NICU (median days: 82 days vs. 47 days, p = 0.0008), more days on mechanical ventilation (medians days: 13 vs. 0, p = 0.0003), and had a statistically significant difference in the five-minute Apgar score between the two groups (7 in the high-risk group vs. 8 in the low-risk group, p = 0.021). Maternal age, antenatal steroids, antenatal magnesium, pre-eclampsia, maternal diabetes, race, and ethnicity were comparable between both groups (Table 1). When infants were stratified by gestational age with each subgroup, given a category and bivariate analysis done between two groups and ANOVA done to compare all groups, 60% of the extremely preterm (22-<28 weeks gestational age/GA), 17% of the very preterm (28-<32 weeks), 17% of the moderate to late preterm (32-<37 weeks), and 6% of the term (≥37 weeks) infants were associated with high ASD risk.

**Table 1:** Characteristics of infants discharged from NICU and assessed for ASD risk by M-CHAT-R/F screening tool NICU: Neonatal intensive care unit; PDA: Patent ductus arteriosus; IVH: Intraventricular hemorrhage; ROP: Retinopathy of prematurity; M-CHAT-R/FL: Modified Checklist for Autism in Toddlers-Revised with Follow-Up; ASD: Autism spectrum disorder.

Characteristics	High ASD risk (N = 35), No. (%)	Low ASD risk (N = 130), No. (%)	P-value
Infant			
Birth weight (kg), median	0.90 (0.49-2.86)	1.18 (0.46-5.21)	0.033
Gestational age (weeks), median	25.6 (23.4-39.0)	29.4 (22.5-40)	0.01
22-<28 weeks	21 (60)	51 (39)	0.028
28-<32 weeks	6 (17)	45 (35)	0.047
32-<37 weeks	6 (17)	14 (11)	0.305
>37 weeks	2 (6)	20 (15)	0.135
Inborn (born in the hospital, not transported)	20 (57)	93 (72)	0.09
Male	20 (57)	78 (60)	0.722
Intubated at birth	20 (57)	58 (45)	0.188
Discharge on O_2_ monitor	14 (40)	29 (23)	0.039
White	18 (51)	61 (47)	0.946
Black	10 (29)	37 (28)	
Hispanic	6 (17)	27 (21)	
Others	1 (3)	5 (4)	
Apgar 1 min (median)	5 (0-9)	5 (0-9)	0.257
Apgar 5 min (median)	7 (2-9)	8 (1-9)	0.021
Mechanical ventilation, days (median)	13 (0-85)	0 (0-95)	0.0003
Length of stay in NICU, days (median)	82 (1-166)	47 (2-370)	0.0008
Postnatal steroids	21 (60)	33 (25)	0.009
PDA	16 (46)	49 (38)	0.389
IVH (all grades)	21 (60)	59 (45)	0.006
Cerebral palsy	9 (26)	4 (3)	<0.0001
Speech delay	20 (57)	40 (31)	0.004
Hearing screen failure at discharge	5 (14)	10 (8)	0.228
Severe ROP	17 (49)	27 (21)	0.012
Maternal			
Maternal age (years) (median)	28 (17-40)	28 (17-46)	0.842
Antenatal steroids	27 (77)	87 (67)	0.246
Antenatal magnesium	17 (49)	61 (47)	0.862
Public insurance	17 (61)	44 (44)	0.184
Pre-eclampsia	2 (6)	17 (13)	0.226
Gestational diabetes	3 (9)	9 (7)	0.739
Singleton birth	8 (26)	26 (20)	0.711
Cesarean delivery	28 (80)	90 (69)	0.210
Maternal psychiatric diagnosis			
Major depression	8 (23)	46 (35)	0.161
Anxiety disorder	12 (34)	49 (38)	0.711
Bipolar disorder	1 (3)	11 (8)	0.257
Schizophrenia	0	2 (1)	0.460

The odds of developing high ASD risk in infants with severe IVH was 1.75 (OR: 1.75, 95% CI: 1.01-3.04) when controlled for gestational age and birth weight (Table 2).

**Table 2:** Risk of ASD positive screen in the infants with IVH controlling for GA and birth weight M-CHAT-R/F: Modified Checklist for Autism in Toddlers-Revised With Follow-Up; ASD: Autism spectrum disorder; IVH: Intraventricular hemorrhage; GA: Gestational age.

	High ASD risk (failed M-CHAT-R/F), No. (%)	OR	95% CI
IVH 1&2 (Mild)	12 (20)	0.32	0.11-0.98
IVH 3&4 (Severe)	9 (43)	1.75	1.01-3.04

Table 3 reports the neurodevelopmental outcomes associated with different grades of IVH. High ASD risk (43% vs. 20%, p = 0.044) and cerebral palsy (19% vs. 5%, p = 0.004) were significantly associated with severe IVH. There were no differences in speech delay, hearing screen failure at NICU discharge, and severe ROP in our cohort of mild and severe IVH infants.

**Table 3:** Intraventricular hemorrhage grades of infants and neurodevelopmental outcomes M-CHAT-R/F: Modified Checklist for Autism in Toddlers-Revised With Follow-Up; ASD: Autism spectrum disorder; IVH: Intraventricular hemorrhage; ROP: Retinopathy of prematurity.

IVH categories	Mild IVH: 59, No. (%)	Severe IVH: 21, No. (%)	P-value
High ASD risk (failed M-CHAT-R/F)	12 (20)	9 (43)	0.044
Cerebral palsy	6 (5)	8 (19)	0.004
Speech delay	28 (22)	9 (21)	0.896
Hearing screen failure at discharge	17 (14)	6 (14)	0.911
Severe ROP	33 (26)	13 (31)	0.781

Table 4 shows a distribution of M-CHAT-R/F screening results with respect to the IVH and no-IVH infants. In relation to infants with IVH and those without IVH, our study displays an imbalance of M-CHAT-R/F screening outcomes. Overall, 26% of infants failed the M-CHAT-R/F compared to 16% of infants in the no-IVH group (p = 0.125). All these findings had no statistical significance. In comparison, the median age at the first M-CHAT-R/F screening was 18 months (17-32 months), and the median age at the follow-up/second screening was 24 months (24-32 months).

**Table 4:** Distribution of M-CHAT-R/F results in IVH and no-IVH groups M-CHAT-R/F: Modified Checklist for Autism in Toddlers-Revised With Follow-Up; ASD: Autism spectrum disorder; IVH: Intraventricular hemorrhage.

M-CHAT-R/F, n	IVH (n = 80), No. (%)	No-IVH (n = 85), No. (%)	P-value
M-CHAT-R/F failed	29 (36)	19 (22)	0.276
M-CHAT-R/F passed	51 (64)	66 (78)	
M-CHAT-R/F failed, then passed on follow-up	8 (10)	5 (6)	
High ASD risk (overall M-CHAT-R/F failed)	21 (26)	14 (16)	0.125

CUS at 36 weeks PMA or discharge from NICU was found in 69 (86%) infants in the IVH group. ASD risk of infants with resolved IVH on CUS was comparable to the no-IVH group (18% vs. 16%). Infants with PVL had 3.24 and those with hydrocephalus had 3.11 odds of developing high ASD risk (OR: 3.24, 95% CI: 0.73-14.34; OR: 3.11, 95% CI: 0.57-16.79). Infants with hydrocephalus needing VP shunt had 4.75 odds of developing high ASD risk (OR: 4.75, 95% CI: 0.73-30.69 (Table 5). These results were also not statistically significant.

**Table 5:** Risk of ASD positive screen in the IVH infants based on the discharge CUS findings CUS: Cranial ultrasound; IVH: Intraventricular hemorrhage; ASD: Autism spectrum disorder; HYDH: Hydrocephalus; VP shunt: Ventriculoperitoneal shunt; PVL: Periventricular leukomalacia; OR: Odds ratio; CI: Confidence interval; M-CHAT-R/F: Modified Checklist for Autism in Toddlers-Revised with Follow-Up.

CUS abnormalities	n	High ASD risk (M-CHAT-R/F failed), n (%)	OR	95% CI
IVH resolved	39	7 (18)	0.78	0.28-2.11
HYDH (no VP shunt)	9	3 (33)	3.11	0.57-16.79
HYDH + VP shunt	7	3 (42)	4.75	0.73-30.69
PVL	14	4 (28)	3.24	0.73-14.34

## Discussion

In this study, we examined the association between ASD risk in infants with IVH along with gestational age, weight at birth, and CUS findings at NICU discharge. ASD risk was assessed using the M-CHAT-R/F screening tool at 16-30 months of chronological age.

ASD screening tests are not diagnostic. They help the providers to identify children who are at risk for ASD or identify other developmental delays that would require additional evaluation and likely benefit from early intervention therapy. Children screened with the M-CHAT-R/F are identified with ASD risk at younger ages than predicted by national statistics [17]. M-CHAT was initially validated in 2001 [15] with a sensitivity of 0.87, a specificity of 0.99, a positive predictive value of 0.80, and a negative predictive value of 0.99. In 2014, it was further validated with the inclusion of follow-up interviews to decrease the number of cases who initially screened positive while maintaining high sensitivity. A meta-analysis [18] by Wieckowski et al. in the year 2023 showed that the pooled sensitivity of MCHAT-R/F was 0.83 (95% CI: 0.77-0.88), and the pooled specificity was 0.94 (95% CI: 0.89-0.97). Previous studies of very preterm infants reported a 21%-41% prevalence of positive M-CHAT questionnaire screens [19]. Unlike the current study, previous reports did not include a term reference group, nor did many studies have a follow-up/second screening interview.

Overall, 26% of the infants in the IVH group screened positive for high ASD risk, 20% in the mild IVH group, 18% in the resolved IVH group, and 16% in the no-IVH group. The incidence is lower than that in prior studies, as anticipated, due to the inclusion of follow-up interviews. Our results also revealed that the incidence of ASD risk is similar in mild IVH, resolved IVH, and no-IVH infants.

While examining the risk factors for screening positive for ASD, we found an inverse and predominant association with gestational age and weight at birth, consistent with previous studies [3,20,21]. When controlled for both gestational age and birth weight, our results have revealed that severe IVH strongly correlates with a high ASD risk and being diagnosed with cerebral palsy. The etiology of ASD in preterm and low birth weight children is poorly understood. Aberrant brain development [7,21,22], reduced brain volume [23], intracranial injuries [24], neonatal pain, and stress [22] are the associated factors described in studies. Like earlier studies, numerous other factors, including low Apgar score [25], cerebral palsy [9], postnatal systemic steroids [26], more days on mechanical ventilation [27], and longer lengths of NICU stay [27], were also found to be associated with the high ASD risk in our study.

Concerning the stability of ASD risk, there have been reports of challenges in identifying the ASD risk earlier among young children. A study reported that few preterm infants classified as having high ASD risk at an early age had little to no ASD concern on later assessment [28]. Another study reported that rates of preterm-born children identified with ASD risk decreased over time and highlighted the importance of repeated assessments for ASD risk. Our data also exhibited similar findings; 13 preterm children classified as having high ASD risk at 18 months were classified as low risk for ASD at 24 months of chronological age. These findings have emphasized the importance of examining the ASD risk over time, particularly in preterm children.

ASD is considered a neurodevelopmental disorder of corticostriatal circuits [29]. A previous study reported that in very preterm infants, CUS abnormalities were associated with higher ASD screening scores on the Social Communication Questionnaire [30]. In our study, infants with discharge CUS findings of PVL or hydrocephalus have demonstrated a threefold increased risk for ASD over those with no evidence of CUS abnormality, which is consistent with a prior study in which the author reported that any white matter injury (ventricular enlargement or parenchymal lesion) tripled the risk for screening positively for ASD compared with no cranial ultrasound abnormalities [7].

Implication of this study

Currently, there is no specific treatment for IVH in newborns who are at risk. Therefore, preventive strategies focused on antenatal management, such as routine corticosteroid administration and magnesium sulfate use; perinatal management, such as maternal transfer to a specialized center; and postnatal management, including prevention of extreme blood pressure, hemodynamic significant PDA management, and optimization of cardiac function and incorporating neuroprotective care bundles in the first 72 hours of life into routine care for such infants may reduce the likelihood of IVH development and its neurodevelopmental and psychological comorbidities such as ASD. This study's results encourage caregivers to implement quality improvement initiatives to decrease the incidence and severity of IVH and support providers in counseling the parents on the risk of ASD in high-risk infants with prematurity, low birth weight, IVH, and abnormal CUS findings. Practitioners should also bear in mind the importance of timely screening for ASD with follow-up screening as recommended by AAP and referrals of high-risk patients for diagnostic confirmation and intervention to improve long-term neurocognitive and developmental outcomes.

Strengths and limitations

The strength of our study is that we included a new version of the M-CHAT-R/F screening tool, which has improved pooled sensitivity (0.83) and pooled specificity (0.94) [18]. Our cohort was not restricted like other preterm studies; we included both preterm and full-term infants. We examined the ASD risk, not only according to gestational age and weight at birth but also based on the severity of IVH and type of brain abnormalities on discharge CUS. IVH is a risk factor, but lower gestational age and birth weight are the more predominant risk factors for ASD.

We realize that our study has a few limitations, such as being a single-center retrospective study. The sample size is small. NICU discharge CUS was not found in 11 (14%) of the infants in the IVH group, either due to practice variation as few providers did not feel the need to do discharge CUS in infants with mild IVH or because it was missed due to being discharged before 36 weeks PMA. Our study’s focus was the ASD risk assessment, given that the M-CHAT-R/F is intended to be used as a screening tool for determining whether further evaluation and intervention are necessary. We did not look at confirmatory diagnostic assessment in the present study and, therefore, were unable to ascertain ultimate ASD diagnosis in those who were screened at high risk for ASD. We did not have an objective developmental tool like Bayley Scales of Infant and Toddler Development (BSID-III) during the study period, which is commonly used in NICU follow-up clinics to assess the neurodevelopment of NICU graduates, which might be a better tool for cognitive, language, motor, socio-emotional, and adaptive behavior assessment.

## Conclusions

Our study indicated that severe IVH, but not mild IVH, increased the risk of ASD and cerebral palsy. Implementation of effective strategies is required to protect against severe IVH and reduce adverse neurological sequelae. Practitioners should also bear in mind the importance of timely screening for ASD with follow-up screening as recommended by AAP and referrals of high-risk patients for diagnostic confirmation and intervention to improve long-term neurocognitive and developmental outcomes.
